# Improving accuracy of medication identification in an older population using a medication bottle color symbol label system

**DOI:** 10.1186/1471-2296-12-142

**Published:** 2011-12-29

**Authors:** Roberto Cardarelli, Christopher Mann, Kimberly G Fulda, Elizabeth Balyakina, Anna Espinoza, Sue Lurie

**Affiliations:** 1 Primary Care Research Center/Texas Prevention Institute, Department of Behavioral and Community Health, University of North Texas Health Science Center at Fort Worth, 3500 Camp Bowie Blvd, Fort Worth, TX 76107, USA; 2 Texas College of Osteopathic Medicine, University of North Texas Health Science Center at Fort Worth, 3500 Camp Bowie Blvd, Fort Worth, TX, 76107, USA; 3 Primary Care Research Center/Texas Prevention Institute, Department of Family Medicine, University of North Texas Health Science Center at Fort Worth, 3500 Camp Bowie Blvd, Fort Worth, TX 76107, USA; 4 Primary Care Research Center/Texas Prevention Institute, University of North Texas Health Science Center at Fort Worth, 3500 Camp Bowie Blvd, Fort Worth, TX 76107, USA; 5 Department of Behavioral and Community Health, University of North Texas Health Science Center at Fort Worth, 3500 Camp Bowie Blvd, Fort Worth, TX 76107, USA

**Keywords:** Medication labeling, medication errors, medication adherence

## Abstract

**Background:**

The purpose of this pilot study was to evaluate and refine an adjuvant system of color-specific symbols that are added to medication bottles and to assess whether this system would increase the ability of patients 65 years of age or older in matching their medication to the indication for which it was prescribed.

**Methods:**

This study was conducted in two phases, consisting of three focus groups of patients from a family medicine clinic (n = 25) and a pre-post medication identification test in a second group of patient participants (n = 100). Results of focus group discussions were used to refine the medication label symbols according to themes and messages identified through qualitative triangulation mechanisms and data analysis techniques. A pre-post medication identification test was conducted in the second phase of the study to assess differences between standard labeling alone and the addition of the refined color-specific symbols. The pre-post test examined the impact of the added labels on participants' ability to accurately match their medication to the indication for which it was prescribed when placed in front of participants and then at a distance of two feet.

**Results:**

Participants appreciated the addition of a visual aid on existing medication labels because it would not be necessary to learn a completely new system of labeling, and generally found the colors and symbols used in the proposed labeling system easy to understand and relevant. Concerns were raised about space constraints on medication bottles, having too much information on the bottle, and having to remember what the colors meant. Symbols and colors were modified if they were found unclear or inappropriate by focus group participants. Pre-post medication identification test results in a second set of participants demonstrated that the addition of the symbol label significantly improved the ability of participants to match their medication to the appropriate medical indication at a distance of two feet (p < 0.001) and approached significant improvement when placed directly in front of participants (p = 0.07).

**Conclusions:**

The proposed medication symbol label system provides a promising adjunct to national efforts in addressing the issue of medication misuse in the home through the improvement of medication labeling. Further research is necessary to determine the effectiveness of the labeling system in real-world settings.

## Background

Medication misuse is a high-priority topic with consequences that result in adverse drug events (ADEs). The Institute of Medicine projects that approximately 1.5 million preventable ADEs occur annually and create a financial burden of approximately $3.5 billion per year in the United States [1]. Among Medicare enrollees 65 years of age and older, the cost of ADEs in ambulatory settings is more than $2 billion, of which $887 million are due to preventable ADEs [2]. The Institute of Medicine estimates that about 530,000 ADEs occur annually among outpatient Medicare patients over the age of 65 and can be a result of forgetting to take medications, taking the wrong quantity, and taking the wrong medication, among other medication adherence related issues [3,4]. Efforts to improve medication safety and delivery have been more readily implemented within hospital and health systems with interventions focused at the practitioner, pharmacy, and nursing staff level [5]. With 2.7 to 3.6 billion outpatient prescriptions dispensed each year, it is critical to develop more focused interventions in the home setting, where the patient is primarily responsible for medication comprehension and adherence [6,7].

Studies have shown that older patients, patients with low health literacy, and those who take multiple medications may have difficulty comprehending medication labels [8-10]. In any given week, 81% of adults take at least one medication with 27% of them consuming at least five different medications [11]. Women over the age of 65 have the highest rate of medication use, with 94% using at least one medication and 57% using five or more. Older patients may also read and comprehend medication labels differently compared to younger populations in terms of reading speed and error rates according to font type [12]. Older adults are particularly vulnerable to experience adverse health outcomes related to medication adherence and comprehension due to higher rates of medication use and lower health literacy than the general U.S. population [13,14].

Important, but limited, measures have been implemented to address medication and patient safety in the home, which include improved and standardized medication labels with clear text, larger font, warning labels, electronic reminder systems and pill boxes [15-17]. A White Paper published by the American College of Physicians Foundation provided a comprehensive review and action plan on patient safety as it pertains to medication bottle labels [18]. One recommendation provided by the White Paper is a need for a symbol system to be incorporated onto medication bottles. The focus of this pilot study was to evaluate and refine such a system of symbols to place on medication bottles and to assess whether this system would increase the accuracy with which patients 65 years of age or older could match their medication to the indication for which it was prescribed.

## Methods

### Development of the Tachygraphic Color Organized Medication System (TCOM)

**Figure 1:**
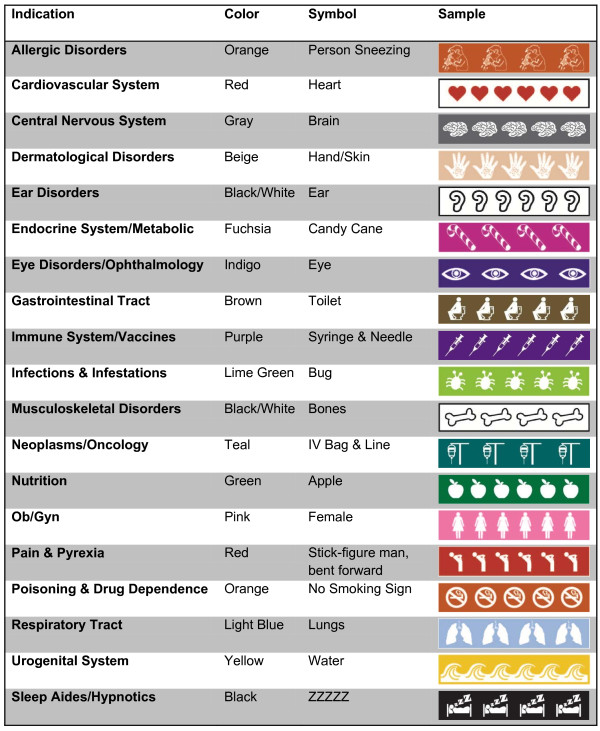
**TCOM medication label system**.

**Figure 2:**
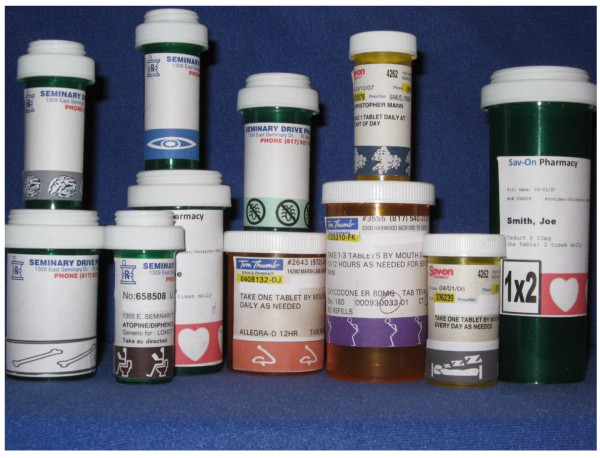
**TCOM sample bottles**.

The TCOM system, also referred to as the medical symbol label system, was conceptualized and developed by researchers at the Primary Care Research Center/Texas Prevention Institute at the University of North Texas Health Science Center at Fort Worth, Texas (UNTHSC). Researchers developed an easily recognizable image (i.e. symbol) and corresponding color for each anatomical main group of the Anatomical Therapeutic Chemical (ATC) classification system recognized by the World Health Organization [19]. The heart symbol and the color red, for example, were used for cardiovascular agents. Some of the 14 main classes were further subdivided (Figure 1). For example, separate labels were created for eye and ear disorders rather than a single label for sensory disorders. This was developed and refined with input and expertise from the medical arts staff of the Informational Technology department at UNTHSC. The labels consist of a series of repeated colored images on a 3/4 inch adhesive strip that can be attached below a standard medication bottle label (Figure 2). Each colored medication symbol strip represents one of 19 common medication indications.

### Study population

The study was conducted in two phases: three focus groups and a pre-post medication identification test. Participants for both phases of the study were recruited from a member clinic of the North Texas Primary Care Practice-Based Research Network (NorTex). NorTex consists of a network of over 130 clinics in the North Texas region and includes primary care clinics focused on family medicine, general internal medicine, pediatrics, geriatrics, and obstetrics/gynecology. A convenience sample was recruited from a family medicine clinic by a coordinator working with clinic staff and physicians. Eligible participants were approached either in the waiting room or immediately after their clinical visit with the physician. During Phase I, participants were scheduled for one of the three focus groups that best met their availability and were consented at the time of the focus group meeting. For Phase II, interested participants were either immediately consented and sent to complete study procedures following their medical visit or were scheduled for a research visit at the NorTex research offices.

Three focus groups were conducted with 7-9 participants each, for a total of 25 participants during the first phase of the study. A separate cohort of 100 participants took part in the pre-post medication identification test portion of the study, Phase II. For both phases, participants were eligible for the study if they were 65 years of age or older and took a minimum of five long-term prescription medications in at least three different medication classes. Individuals with vision loss were excluded from participating based on the visual nature of the study. All study participants were English speaking. Study procedures were approved by the UNTHSC Institutional Review Board.

### Study Procedures

Focus groups were used to provide feedback on the preliminary medication symbol system for 19 medication indications [20]. A trained facilitator conducted focus groups using a developed focus group moderator guide. Focus groups took place at the Primary Care Research Center at the University of North Texas Health Science Center, and each participant was reimbursed $30 for their time and effort. Each focus group lasted approximately 90 minutes, and the conversations were documented through digital audio recording and note taking. A trained individual transcribed all audio recordings. Focus group results were used to modify the medication labeling system according to participant recommendations which were implemented prior to the second phase of the study.

A pre-post medication identification test was conducted in Phase II to assess differences between standard medication bottle labeling alone and the addition of the TCOM medication label symbols in terms of participants' ability to accurately match their prescribed medication to the correct indication. Participants were asked to bring all of their medications, including over-the-counter products, to the study visit. Each participant was asked a series of questions related to identifying each medication, its associated dosage, and the medical conditions for which their medications were prescribed. Participants were then asked to match their medication bottles with the appropriate medical condition. A TCOM label for the corresponding medical indication was then added to the bottom of each bottle below the existing pharmacy medication label, and the participant was again asked to match each bottle with the appropriate medical condition. This procedure was repeated from a distance of two feet, representing the distance needed to identify a medication bottle from common medication storage locations such as kitchen counters, medicine cabinets, cupboards or dressers. Each study visit took approximately 30 minutes, and participants were reimbursed $20 for their time and effort.

### Qualitative measures and analysis

Focus group data were analyzed using thematic content analysis. Data were collated on knowledge of the purpose of medication labeling, barriers in current labeling systems, initial thoughts on the TCOM system, and potential barriers and benefits of the TCOM system. The responses to each question were compiled for a total of 25 participants for the three focus groups. Themes or codes were then derived from all of the responses to each question. These qualitative results were then used to modify and refine the proposed labeling system prior to the commencement of the second phase of the study.

### Quantitative measures and analysis

During the Phase II pre-post medication identification test, data were collected on the number of medications a participant was taking, the number of medications the participant could correctly match with its prescribed indication before the label was added, the number of medications a participant could correctly match with its prescribed indication after the label was added, and the number of medications the participant could correctly match with its prescribed indication two feet away from the medication bottles pre and post labeling.

A power analysis was conducted for Phase II of the study based on 100 participants. Using a paired samples t-test and alpha of 0.05, a sample size of 100 allowed greater than 80% power to detect a difference. Calculations assumed participants would incorrectly identify at least one medication in the pre-measurement and would correctly identify all medications in the post-measurement. Since a minimum of five medications was required for inclusion in the study, this constituted a 20% difference.

The number of medications a participant identified correctly out of total medications was converted to a percent correct, or score, for both the pre- and post-measurements. Due to a non-normal distribution and the paired association of pre and post scores, a Wilcoxon Signed Ranks Test was performed to examine differences between pre and post scores for identifying the medication bottles while in front of the person and at two feet away. Differences were considered statistically significant with a two-tailed probability of 0.05 or less.

## Results

### Phase I results

When asked about knowledge of medication labels, focus group participants commonly identified information and instructions found on standard labels such as when to take medicine, frequency, and strength of medication. Participants expressed that additional information is needed on current medication labels such as the purpose of the medication, and the color/size of pills. They indicated that the writing was typically too small to read, too much information was placed on the label, and that color coding would be helpful unless someone was color-blind. Participants stated that clear instructions on medication schedule and dosage, larger type, and bold letters were needed, especially for those who were visually-impaired. Similarities in size or color of different medications and change by manufacturers in size or color of pills were identified as barriers to medication recognition. Participants also stated that medications could be confusing to identify if they were similar in appearance to other medications, name-brand versus generic, or contained unclear, inadequate instructions. Medication cost also emerged as a theme and important concern for participants. Participants reported that Medicare only covers generic medications, forcing patients to shop around to try to find the best price, and some individuals split pills to save money.

Participants stated that a new symbol label system would be beneficial and appreciated the idea of an additional aid incorporated with existing medication labels because it would not be necessary to learn a completely new system of labeling. Participants generally found that the colors and symbols used in the proposed labeling system were easy to understand, relevant, and "self-explanatory." They noted that colors and symbols were useful for both English and non-English speakers, and found certain colors such as black for sleep aids or blue for respiratory medications particularly relevant.

Concerns were raised about having too much information on the bottle, not having enough space for the TCOM label in addition to existing labeling, and having to remember what colors meant. Participants stated that some colors were inappropriate and unclear in relation to the associated indication, and some symbols were confusing and unfamiliar. Participant concerns and suggestions were then used to revise the labeling system. For example, the color red was incorporated into the symbol for pain and pyrexia because participants associated the color red more closely with pain than the original purple color used in the symbol. Participants also suggested that colors should be bright and should be placed either at the bottom of the bottle or added to printed inserts on side effects.

### Phase II results

Table 1 provides the demographic characteristics of the 100 participants of Phase II. One participant did not complete the post label questions; therefore, that participant is excluded from the paired analysis. The participants were 56% male, 82% white, and 67% had more than a high school education. The average age of participants was 73.4 years (range 65-88 years). Participants took an average of 11 different medications (range 5-29) from 8 different classifications (range 3-17).

**Table 1:** Demographic Characteristics of the TCOM Sample (n = 100)

Variable	n	(%)
Gender		
Male	56	(56.0)
Female	44	(44.0)

Race/Ethnicity		
White/Caucasian	82	(82.0)
Black/African American	12	(12.0)
Hispanic	5	(5.0)
Other	1	(1.0)

Highest Grade Completed*		
Less than High School	8	(8.0)
High School	24	(24.0)
More than High School	67	(67.0)

	**Mean**	(sd)**

Age	73.4	5.6
Number of Medications	11.4	5.1
Number of Medication Classes	7.7	2.9

*Highest grade completed is missing for one respondent.

** sd = standard deviation

Prior to placing the TCOM label on the medication bottles, 88.6% of the medications were correctly matched to their prescribed indication when placed in front of participants (Table 2). Only 81.1% of the medications were identified correctly when placed at a 2-feet distance. After placing the TCOM label onto medication bottles, the percent identified correctly significantly improved when bottles were placed at a 2-feet distance (88.6%, p < 0.001) and trended toward significance when placed in front of participants (92.3%, p = 0.07). An effect size (Cohen's d) of 0.15 was obtained for medications placed in front of participants and 0.25 for medications placed at two feet away.

**Table 2:** Percent of Medication Bottles Identified Correctly

Variable	Average Percent Correct	p-value*
In front of person pre label	88.5	0.07
In front of person post label	92.3	

At two feet distance pre label	81.1	< 0.001
At two feet distance post label	88.6	

*Wilcoxon Signed Rank Test

## Discussion

Although institutional and policy level changes have been implemented to improve medication adherence, [21-24] less attention has been placed on elderly consumers' ability to understand medication labels to reduce medication misuse at home [18]. The development of an easily implementable tool such as the TCOM labeling system offers pharmacies and health care providers the potential to decrease medication errors among adults 65 years of age and older, a population that is more likely to be on multiple medications and is more vulnerable to adverse drug events than the general population [25,26]. The TCOM system can empower older adults with an accessible system to enhance correct use of medications in the home and potentially decrease adverse events associated with medication misuse or incorrect use.

Qualitative responses by study participants confirmed the need for clear label formatting, explicit instructions on dosage, and supported the idea of adding an easily identifiable visual aid to existing medication labels [18]. Hwang and colleagues found that commonly used illustrations that are currently used on medication labels do not increase patients' ability to identify their medications, and in some cases, may be ambiguous or misleading [27]. Although further refinement of our TCOM label system may be necessary to ensure the clarity of the colors and symbols used, our pilot study implemented a focus group phase to foster input into the development of a set of symbols that would be easily identifiable by the target population. Pre-post medication identification test results show that the TCOM label system did in fact improve patients' ability to correctly identify their medications from a distance of two feet, and approached significance when placed directly in front of study participants. These results suggest that the proposed labeling system has the potential to be a helpful visual addition to current medication bottle labels, since visual aids currently used on bottle labels may not be effective.

Although participants were able to more accurately identify their medications at a distance of two feet after the addition of the TCOM label, the small effect size may call into question the clinical relevance of study results. However, the incorrect use of even one medication can result in adverse events, including hospitalizations or deaths, and/or suboptimal treatment of a medical condition. Therefore, even small improvements in medication identification have the potential to reduce the incidence of preventable adverse drug events and improve medical outcomes. Care must also be taken in the interpretation of study results due to the small sample size. Results are specific to patients 65 and older from a member clinic of the North Texas Primary Care Practice-Based Research Network and may not be generalizable to other populations. Recall bias may have occurred during the implementation of the pre-post test assessments. Participants may have remembered the purpose of their medications more clearly after the addition of the TCOM label since they were asked to recall the purpose of their medicines prior to the addition of the label. However, participants were not informed whether or not they had matched their medication to the correct indication during the assessment process, and they were expected to already be familiar with their medications. Participants were not asked to learn a set of new indications, but rather were required to be currently taking a minimum of five prescription medications in at least three medication classes in order to qualify for the study.

It is difficult to assess the impact of recall bias on study results due to lack of a comparison group and randomized study design. To address this issue, further study is recommended to determine the utility of a visual system of labeling in real-world settings to assess the comparative effectiveness of the proposed labeling system to current bottle labels. Studies have shown that adults 65 years of age and older have the lowest average literacy rate among all age groups and that literacy can have a significant impact on a patient's ability to recognize medications [9,28]. Further study is needed in home, pharmacy, and community settings to determine how social factors outside of a research setting impact the suitability and effectiveness of the TCOM labeling system.

## Conclusions

Pilot study results show a promising addition in efforts to address medication misuse in the home through the improvement of medication labeling. Further research is necessary in order to determine the effectiveness of the TCOM labeling system in real-world settings.

## Abbreviations

TCOM: Tachygraphic Color Organized Medication label system; ADE: adverse drug event; NorTex: North Texas Primary Care Practice-Based Research Network; ATC: Anatomical Therapeutic Chemical Classification System.

## Competing interests

The authors declare that they have no competing interests.

## Authors' contributions

RC and CM conceived the study, developed the design and were the principal investigators of the study. RC and EB were the primary writers of the manuscript. KGF assisted in methodology of the study, conducted data analyses, and edited the manuscript. AE oversaw the daily activities of the study and edited the manuscript. SL conducted the focus groups and performed analysis for the study and also reviewed and edited the manuscript. All authors read and approved the final manuscript.

## Pre-publication history

The pre-publication history for this paper can be accessed here:

http://www.biomedcentral.com/1471-2296/12/142/prepub
